# Genome characterisation of the genus Francisella reveals insight into similar evolutionary paths in pathogens of mammals and fish

**DOI:** 10.1186/1471-2164-13-268

**Published:** 2012-06-22

**Authors:** Andreas Sjödin, Kerstin Svensson, Caroline Öhrman, Jon Ahlinder, Petter Lindgren, Samuel Duodu, Anders Johansson, Duncan J Colquhoun, Pär Larsson, Mats Forsman

**Affiliations:** 1 Division of CBRN Security and Defence, FOI - Swedish Defence Research Agency, Umeå, Sweden; 2 Section for Bacteriology, Norwegian Veterinary Institute, Postbox 750 sentrum, 0106, Oslo, Norway; 3 Department of Clinical Microbiology, Umeå University, SE-901 85, Umeå, Sweden

**Keywords:** *Francisella*, Next-generation sequencing, Recombination, Fish, Genetics, Evolution

## Abstract

**Background:**

Prior to this study, relatively few strains of *Francisella* had been genome-sequenced. Previously published *Francisella* genome sequences were largely restricted to the zoonotic agent *F. tularensis*. Only limited data were available for other members of the *Francisella* genus, including *F. philomiragia*, an opportunistic pathogen of humans, *F. noatunensis,* a serious pathogen of farmed fish, and other less well described endosymbiotic species.

**Results:**

We determined the phylogenetic relationships of all known *Francisella* species, including some for which the phylogenetic positions were previously uncertain. The genus *Francisella* could be divided into two main genetic clades: one included *F. tularensis, F. novicida, F. hispaniensis* and *Wolbachia persica*, and another included *F. philomiragia* and *F. noatunensis*.

Some *Francisella* species were found to have significant recombination frequencies. However, the fish pathogen *F. noatunensis* subsp. *noatunensis* was an exception due to it exhibiting a highly clonal population structure similar to the human pathogen *F. tularensis.*

**Conclusions:**

The genus *Francisella* can be divided into two main genetic clades occupying both terrestrial and marine habitats. However, our analyses suggest that the ancestral *Francisella* species originated in a marine habitat. The observed genome to genome variation in gene content and IS elements of different species supports the view that similar evolutionary paths of host adaptation developed independently in *F. tularensis* (infecting mammals) and *F. noatunensis* subsp. *noatunensis* (infecting fish).

## Background

Tularemia is a zoonotic disease caused by the highly infectious, virulent, Gram-negative bacterium *Francisella tularensis*. Due to its infectious nature, ease of dissemination and associated high fatality rate (especially from respiratory infection), *F. tularensis* has been included in military biological weapons programs and is regarded as one of the top six agents which have a high potential for misuse in bioterrorism [1].

The known diversity of the genus *Francisella* has recently expanded substantially and the boundaries between taxonomic units within the genus is currently under debate [2,3]. In mammals, including man, the disease tularemia is caused by two *F. tularensis* subspecies: subsp. *tularensis* (type A, endemic in North America) and subsp. *holarctica* (type B, found throughout the Northern hemisphere) [4]. Nearly all fatalities due to tularemia are caused by *F. tularensis* type A infections, for which mortality rates up to 30% have been reported [5]. In contrast, *F. tularensis* type B causes a milder disease, for which fatalities rarely occur. A third accepted subspecies, *F. tularensis* subsp. *mediasiatica* is found in central Asia. This subspecies has been found to display level of virulence comparable to strains of subspecies *holarctica* and very rarely cause human infections [6,7]. Disease in humans has also been presented following opportunistic infection by *F. novicida* (in individuals with a weakened immune system) and *F. philomiragia* (in individuals with a weakened immune system exposed to sea water) [8-10].

A number of *Francisella* lineages also exist that do not appear to be associated with human disease. Strains closely related to *F. philomiragia*, which have recently been designated as *Francisella noatunensis,* have been identified as etiological agents of Francisellosis in fish [11-15]. *F. noatunensis* is further divided into two subspecies, subsp. *noatunensis*[16] and subsp. *orientalis*[17,18]. Recent published studies have also identified several new related and as yet uncultured forms of *Francisella* from soil and water [19-23], as well as tick endosymbionts with high similarity to *F. tularensis* (*Francisella*-like endosymbionts) [24]. One isolated tick endosymbiont has inaccurately been designated *Wolbachia persica*[25], incorrectly placing it within the *alpha-*proteobacteria [26]. Further, a marine ciliate endosymbiont has been proposed to represent ‘*Candidatus F. noatunensis* subsp. *endociliophora*’ [23] and a bacterium isolated from an air-conditioning system has been tentatively classified as *Francisella cantonensis*[27]. Thus, in recent years the existence of substantial sub-species diversity within the *Francisella* genus has become increasingly apparent. Members of *Francisella* appear to be associated with a multitude of different ecological niches, ranging from specialised endosymbionts and pathogens with different host spectrums, to generalists believed capable of a free-living existence.

There are several reasons for sequencing *Francisella* strains other than those belonging to *F. tularensis*. Analysis of gene content and genetic relationships may help to improve our understanding of the biology and evolution of these bacteria, including that of the human pathogen *F. tularensis.* Sequencing multiple strains of environmental *Francisella* may also reveal important sequence differences between environmental species and *F. tularensis*. This would allow the design of increasingly specific *F. tularensis* detection assays for use in anti-bioterrorism applications and epidemiological studies, particularly important since current methods frequently falsely detect *F. tularensis* in environmental samples [28].

The genome sequence data generated by the present study represents a considerable advancement of knowledge of the genus *Francisella* and includes genome sequences for 18 environmental *Francisella* strains, four *F. tularensis* and two distant but genetically related species *Fangia hongkongensis*[29] and *Piscirickettsia salmonis*[30]*.* Here we describe the population structure of the genus *Francisella*, suggest that ancestral *Francisella* strains originated in marine habitats, show that present-day strains can be divided into two main genetic clades occupying continental and aquatic habitats and provide evidence that similar evolutionary paths of host adaptations have occurred independently in *F. tularensis* (infecting mammals) and *F. noatunensis* subsp. *noatunensis* (infecting fish).

## Results

### Bacterial strains and classifications

Here, we present 24 novel genome sequences representing all major subclades of the *Francisella* genus (Table 1), with the exception of the recently discovered *F. cantonensis*[27]. The data doubles the number of published *Francisella* genomes and significantly extends the known range of genetic diversity. The studied material includes seven previously unsequenced *F. noatunensis* isolates and a single representative from the tick endosymbiont group [31]. In addition, representatives of *Fa. hongkongensis*[29] and *P. salmonis*[32]*,* both of which are related to the genus *Francisella*, were also sequenced. Interestingly, *Francisella* strain 3523 [33,34] appeared to belong to the *F. hispaniensis* clade and not the *F. novicida* clade as reported previously [34].

**Table 1:** Strains sequenced in this study

Species	FSC ID	Alternate designation	Year isolated	Source	Location	NCBI ID
*F. tularensis subsp. holarctica*	FSC021	F0014, Tsuchiya	1958	Human lymph node	Fukushima prefecture, Japan	73369
*F. tularensis subsp. holarctica*	FSC208	3049UBG	1998	Human	Gävleborg county, Sweden	73467
*F. tularensis subsp. holarctica*	FSC539	2004-32-55	2004	Human blood	Örebro county, Sweden	73393
*F. tularensis subsp. mediasiatica*	FSC148	F0012, 240, 840	1982	Tick	Jambyl oblast, Kazakhstan	73379
*F. tularensis subsp. tularensis*	FSC054	F0010, Nevada 14	1953	Hare (*Lepus californicus*)	Nevada state, USA	73375
*F. novicida*	FSC159	F0052; fx2; 110	1995	Human blood	Texas state, USA	73383
*F. novicida*	FSC160	fx1; Houston, 2766	1991	Human blood	Texas state, USA	73385
*F. novicida*	FSC595	F58	2003	Human blood	Brazil/UK/Germany	73395
*F. hispaniensis*	FSC454	FhSp1	2003	Human blood	Elche, Spain	73391
*F. noatunensis subsp. noatunensis*	FDC178	NVI 7601, F/134/09A	2009	Atlantic cod (*Gadus morhua*)	Waterford, Ireland	73465
*F. noatunensis subsp. noatunensis*	FSC769	NVI 2005/50/F292-6C, NCIMB14265, LMG23800, DSM12596	2005	Atlantic cod (*Gadus morhua*)	Hordaland county, Norway	73397
*F. noatunensis subsp. noatunensis*	FSC772	NVI 5888, PQ1106	2006	Atlantic salmon (*Salmo salar*)	Lake Llanquihue, Chile	73449
*F. noatunensis subsp. noatunensis*	FSC774^2^	NVI 5865, GM2212	2004	Atlantic cod (*Gadus morhua*)	Rogaland county, Norway	73457
*F. noatunensis subsp. noatunensis*	FSC775^2^	DSM18777, GM2212	2004	Atlantic cod (*Gadus morhua*)	Rogaland county, Norway	73459
*F. noatunensis subsp. noatunensis*	FSC846	NVI 5518, SVA 74/04	2004	Atlantic cod (*Gadus morhua*)	Southern Skagerrak, Sweden	73463
*F. noatunensis subsp. orientalis*	FSC770	NVI 5409, PQ1104,	2006	Tilapia (*Oreochromis* sp.)	Costa Rica	73389
*F. noatunensis subsp. orientalis*	FSC771	Ehime-1, PQ1105	2001	Three-line grunt (*Parapristipoma trilineatum*)	Uwajima, Ehime prefecture, Japan	73447
*Wolbachia persica*	FSC845	ATCC VR - 331	1960	Soft tick (Oken) (*Argas persicus*) from buff-backed heron (*Bubulcus ibis*)	Nile Barrage Park, Cairo, Egypt	73171
*F. philomiragia*	FSC037	F0047, ATCC 25016	1960	Water	Bear River Refuge, Utah state, USA	73371
*F. philomiragia*	FSC039	F0049, ATCC 25018	1960	Water	Odgen Bay Refuge, Utah state, USA	73373
*F. philomiragia*	FSC145	F0046; CCUG 12603	1982	Human abscess	Göteborg, Sweden	73377
*F. philomiragia*	FSC154	Swiss	1979	Bone marrow, ascitic fluid	Zürich, Switzerland	73381
*Fangia hongkongensis*	FSC776	JCM14605	2004	Seawater at the outlet of a sand filter	Port Shelter, Hongkong, China	73461
*Piscirickettsia salmonis*	FSC773	NVI 5692	2006	Atlantic salmon (*Salmo salar*)	Møre & Romsdal county, Norway	73451

1 FDC178 was supplied by the Marine Institute, Oranmore, Ireland.

2 FSC774 was supplied by the National Veterinary Institue (NVI), Norway and FSC775 was requisitioned from the German Microorganism Collection (DSM).

3 FSC 160 (fx1) was supplied by VAMC, Houston, Texas. (Previously published FSC156 (fx1) was supplied by another lab.).

4 FSC539 from same patient as FSC529 in Svensson et al 2009.

Information about isolates sequenced in this study.

**Figure 1:**
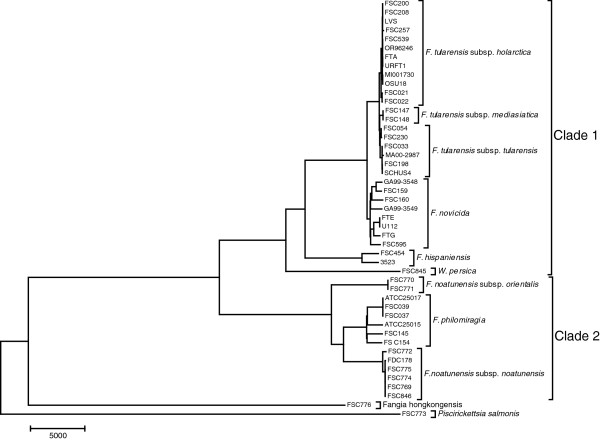
**Whole-genome phylogeny.** The phylogeny of 45 *Francisella* strains and two genetic relatives, *Fangia hongkongensis* and *Piscirickettsia salmonis,* based on single nucleotide polymorphism in the core genome. The *Francisella* genus is divided into two parts, clade 1 (*F. tularensis* and *F. novicida*) and clade 2 (*F. noatunensis* and *F. philomiragia*).

Overall, a major bifurcation into two genetic lineages was identified (Figure 1) with clade 1 comprising *F. tularensis, F. novicida, F. hispaniensis* and *W. persica*, and clade 2 containing *F. noatunensis* and *F. philomiragia*.

### *Francisella* whole-genome phylogeny and average nucleotide identities

Whole-genome alignments were produced using progressiveMauve [35]. A phylogeny based on core genome single nucleotide polymorphisms is shown in Figure 1.

Analysis of the whole-genome phylogeny showed that strains of *F. philomiragia* and *F. noatunensis* subsp. *noatunensis* formed sister clades, while strains of *F. noatunensis* subsp. o*rientalis* diverged at a more basal position, indicating the newly named species *F. noatunensis* is paraphyletic. This interpretation was supported by an analysis of average nucleotide identities (ANI): ANI for *F. philomiragia* compared to *F. noatunensis* subsp. *noatunensis* is higher (95%) than *F. philomiragia* compared to *F. noatunensis* subsp. *orientalis* (92–93%) Additional file 1.

Phylogenetic inference using whole-genome data and 16 S ribosomal RNA genes resulted in incongruent topologies. The phylogenetic position of *F. philomiragia* (Figure 1) was different from previous publications of phylogenies calculated based on 16 S ribosomal RNA genes (Additional file 2) [13,14].

### Analysis of *Francisella* pan and core genomes

**Figure 2:**
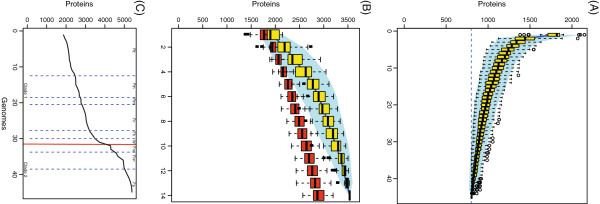
**Overview of core and pan genome structure for the *Francisella *genus.A**. Accumulation curve (core genome) for the number of genes in common as a function of the number of genomes analysed for the *Francisella* genus. **B**. Accumulation curve for the total number of genes (pan genome) as a function of the number of genomes analysed for clade 1 (*F. tularensis, W. persica and F. hispaniensis*)(red) and clade 2 (*F. philomiragia* and *F. noatunensis*)(yellow). **C**. Cumulative accumulation of total number genes when genomes are added according to the structure in the SNP phylogeny. Ftt – F. tularensis subsp. tularensis, Fth – *F. tularensis subsp. holarctica*, Ftm – F. *tularensis subsp. mediasiatica*, Fn – *F. novicida*, Fh – *F. hispaniensis*, W – *Wolbachia persica*, Fno – *F. noatunensis* subsp. *orientalis*, Fnn – *F. noatunensis* subsp*. noatunensis*, Fp – *F. Philomiragia.*

Based on the analysed gene orthologs, the core genome of the genus *Francisella* contained 803 genes (Figure 2A). Clade 1 shared 854 genes, while clade 2 (*F. noatunensis* and *F. philomiragia*) shared 1359 genes (Figure 2B).

The pan-genome of the genus *Francisell*a contained a total of 5396 genes (Figure 2C). Separate analysis of the pan-genome size within clade 1, excluding *F. hispaniensis and W. persica*, and clade 2 identified 3222 and 3535 genes, respectively (Figure 2B). The pan-genome of *F. tularensis* contained 2769 genes.

### Clade-specific genes

**Figure 3:**
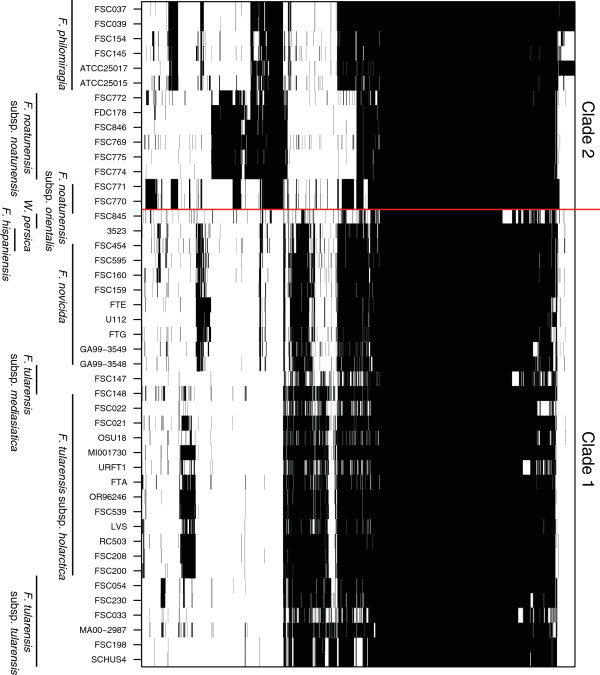
**Whole-genome comparison of the pan-genome of 45 *Francisella *strains.** Presence of a gene in a genome is indicated in black. The core genome of 803 genes, and the genes unique to clade 1 (*F. tularensis*, *F. novicida, F. hispaniensis and W. persica*) and clade 2 (*F. noatunensis* and *F. philomiragia*) are clustered together in the heat map.

An overview of the gene distribution in the genus *Francisella* is depicted in Figure 3 and the clade-specific genes are summarised in Table 2. The human pathogen*, F. tularensis* contained only seven unique genes (FTT0794, FTT0795, FTT0796, FTT1188, FTT1453c, FTT1454c and FTT1458), which grouped at three different locations in the genome. No unique genes were found in *F. novicida* genomes. *F. hispaniensis*, *F. noatunensis* and *F. philomiragia* exhibited seven, nine and ten unique genes, respectively. The complete clade 2 (*F. noatunensis* and *F. philomiragia*) genomes displayed 41 genes that were not present in clade 1. However, the clade 2 species *F. noatunensis* subsp. *noatunensis* and *F. noatunensis* subsp. *orientalis* shared 32 and 114 genes, respectively, which were not present in other genomes.

**Table 2:** Clade-specific genes

**Included species/subspecies**	**Excluded species/subspecies**	**#unique genes**	**Reference genome**
*F. tularensis subsp. tularensis*	All other	0	SCHUS4
*F. tularensis subsp. holarctica*	All other	0	LVS
*F. tularensis subsp. mediasiatica*	All other	0	FSC147
*F. philomiragia*	All other	9	ATOC 25017
*F. noatunensis subsp. noatunensis*	All other	32	FSC769
*F. noatunensis subsp. orientalis*	All other	114	FSC770
*F. novicida*	All other	0	U112
*F. hispaniensis*	All other	7	FSC454
*Wolbachia persica*	All other	125	FSC845
*Fangia hongkongensis*	All other	1104	FSC776
*Piscirickettsia salmonis*	All other	1725	FSC773
*F. tularensis subsp. tularensis, F. tularensis subsp. holarctica, F. tularensis subsp. mediasiatica*	F. novicida, F. hispaniensis, W. persica F. noatunensis subsp. orientalis, F. noatunensis subsp. noatunensis, P salmonis	7	SCHUS4
*F. noatunensis subsp. orientalis, F. noatunensis subsp. noatunensis, F. philomiragia*	F. tularensis subsp. tularensis, F. tularensis subsp. holarctica, F. tularensis subsp. mediasiatica, F. novicida, F. hispaniensis, W. persica, F. hongkongensis, P. salmonis	41	FSC769
*F. noatunensis subsp. orientalis, F. noatunensis subsp. noatunensis*	F. philomiragia, F tularensis subsp. tularensis, F. tularensis subsp. holarctica, F. tularensis subsp. mediasiatica, F. novicida, F. hispaniensis, W. persica, F. hongkongensis, P.	10	FSC769
*F. noatunensis subsp. noatunensis, F. philomiragia*	F. noatunensis subsp. orientalis, F. tularensis subsp. tularensis, F. tularensis subsp. holarctica, F. tularensis subsp. mediasiatica, F. novicida, F. hispaniensis, P. salmonis	9	FSC769

Summary of clade-specific genes in the *Francisella* genus.

### IS elements

Notable differences in IS element content were identified between different species and clades. In clade 1, *F. hispaniensis* contained a few copies of ISFtu2, ISFtu3, ISFtu4, ISFpi1 and IS4502, while *W. persica* contained very few copies of ISFtu2 and ISFtu3. The isolates in clade 2 exhibited a different pattern of IS elements. Whereas *F. noatunensis* subsp. *orientalis* was almost completely lacking IS elements (only a potential trace of ISFpi1 was found), *F. noatunensis* subsp. *noatunensis* exhibited an expansion trend similar to that previously reported for *F. tularensis*[36]. The expansion was within the ISFpi1 group and included a handful of copies of the IS4502 element, and single copies of ISFtu1, ISFtu2 and ISFtu3 elements.

The more distant relatives studied, i.e. *Fa. hongkongensis* and *P. salmonis,* contained completely different sets of IS elements compared with *Francisella*. The genome of *P. salmonis* was enriched with ISPsa1 and ISPsa2 [37], whereas only one copy of ISFpi1 was found in *Fa. hongkongensis.* The IS element distributions of *F. tularensis*, *F. novicida* and *F. philomiragia* have been reported previously [36,38].

### Recombination

**Figure 4:**
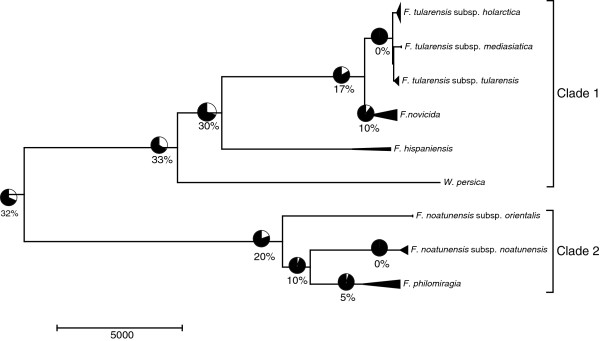
**Overview of the recombination.** The proportion of genes showing evidence of recombination overlaid on the *Francisella* phylogeny. Recombination detection was performed using the PHI method with the number of significant tests (*P* < 0.01) reported as a percentage of recombinant genes within the *Francisella* genus.

Results of the recombination analyses are presented in Figure 4 and Additional file 3. The proportion of genes within the *Francisella* core genome demonstrating evidence of recombination was 32%, although the presence of recombination was highly variable across the different *Francisella* lineages. For some species/subspecies, recombination was entirely absent (i.e. *F. tularensis* subsp. *tularensis, F. tularensis* subsp. *holarctica* and *F. noatunensis* subsp*. noatunensis*), whereas recombination in *F. philomiragia* and *F. novicida* occurred frequently.

## Discussion

To date, sequenced *Francisella* genomes have mainly been of the human pathogenic species *F. tularensis.* Consequently, conclusions about evolutionary processes within the genus *Francisella* have largely been based on *F. tularensis* genomes [36,38].

Moreover, a number of identification assays developed on the basis of *F. tularensis* genome sequences have been shown to give false-positive results. These have been attributed to the presence of the same sequence signatures as in other environmental *Francisella* lineages [39].

In the present study, we provide details of the genetic content of nearly all currently known members of the genus *Francisella*, with the exception of the recently published *F. cantonensis*[27]*.* Despite a generally high sequence similarity within the genus, we found that different genetic clades demonstrated different population structures (recombination frequencies). This suggests differences in lifestyle and host association.

*F. tularensis* has been found to be a highly clonal species, differentiating it from environmental lineages characterised by moderate recombination rates. We also found evidence that the fish pathogen *F. noatunensis* subsp. *noatunensis* also possesses a highly clonal lineage. The low recombination frequency in *F. noatunensis* subsp. *noatunensis* may indicate that a close host association reduced contact with other *Francisella* species, and thereby, lowered opportunities for gene acquisition by recombination, as previously hypothesised for *F. tularensis*[36]. The data of population structure and variability observed among *F. novicida* opposed to *F. tularensis* subspecies *tularensis*, *holarctica* and *mediasiatica* strains should be taken in consideration for the on-going discussion whether it should be a separate species or included as a subspecies in *F. tularensis*.

### Whole genome phylogeny

Our analyses of 45 *Francisella* genomes showed that recognised species could be divided into two main genetic clades (Figure 1) and suggested that the pathogenic species in each clade emerged independently by similar evolutionary paths of host adaptation. *F. tularensis*, which can infect mammals, belonged to clade 1, whereas *F. noatunensis* subsp. *noatunensis,* which infects fish, belonged to clade 2. Our analyses based on the phylogeny of *Francisella* genomes and genetic relatives suggest that the ancestral *Francisella* species originated in a marine habitat.

Using whole-genome analysis, we found evidence that *F. noatunensis* subsp. *noatunensis* and *F. noatunensis* subsp. *orientalis* represent a paraphyletic group. *F. philomiragia* and *F. noatunensis* subsp. *noatunensis* together form a sister clade to *F. noatunensis* subsp. *orientalis* (Figure 1, Table 1). This result was not supported by 16 S RNA analysis, which suggested that the two subspecies *F. noatunensis* subsp. *orientalis* and *F. noatunensis* subsp. *noatunensis* form a clade. There are limited nucleotide differences in the 16 S region and more comprehensive core genome SNP analysis gives a better view of the real relationship. Identification of genetic factors coding for fish pathogenicity may therefore be possible via comparative analyses. Further phenotypic studies and experiments are needed to confirm this hypothesis. Since *F. noatunensis* subsp. *noatunensis* is isolated from cod and salmon, while *F. noatunensis* subsp. *orientalis* isolates are recovered from tilapia and three-line grunt, an alternative hypothesis is that these subspecies evolved their abilities for fish infection independently.

In agreement with other studies [26,40], our analyses verified the phylogenetic position of the tick endosymbiont *W. persica* within the *Francisella* genus. Several lines of genomic evidence support the view that this organism adapted as a primary symbiont; its genome size was found to be significantly less (about 80–85%) than other *Francisella* genomes. Moreover, in *W. persica,* regulatory genes were found to be depleted, while biosynthesis pathways supplying amino acids to their insect host were retained, and there was a low abundance of IS elements. Our results confirm that *W. persica* and other *Francisella*-like endosymbionts (FLE) [19,24,41,42] belong to the genus *Francisella* and that *W. persica* has undergone a significant reduction in its genome.

An important outcome of the present study was reclassification of the isolate 3523 from Australia. It was initially classified as an unusual *F. novicida*[33], a classification which remained despite whole genome sequencing [34]. It was clear from the present analyses that isolate 3523 belongs to the *F. hispaniensis* clade [43]. This result demonstrates the importance of broad-scale intra-genus sequencing for correct classification of new isolates.

### *Francisella* pan- and core- genome

The overall core genome size of 799 genes for the genus *Francisella* was similar to that reported for the genus *Bacillus* (Figure 2A) [44]. Bacterial pan-genomes at the genus level have only recently been reported in the literature, and the number of gene families in these genera is expected to grow as more genomes are included. This approach may be valuable in revealing genes specific for niche commonalities between subspecies.

We detected different pan-genome sizes for clade 1 and clade 2 (Figure 2B), where clade 2 contain more genes compared to clade 1 and especially *F. tularensis* clade alone. As a result, *F. tularensis* may be fully sampled by sequencing smaller set of representative isolates, whereas more isolates of clade 2 would need to be fully sequenced to give a true pan-genome size.

The contribution of each genome to the complete pan-genome of the genus *Francisella* and related organisms is illustrated in Figure 2C. Most genomes contribute to the increase of the pan-genome, although the increase is less pronounced when similar genomes are added.

### Clade specific genes

Genomes in the *Francisella* genus have, with the exception of *W. persica*, similar gene composition. *W. persica* has not retained genes included in the COG group ‘amino acid transport and metabolism’, which it does not require because of its endosymbiotic lifestyle.

An extensive list of 80 potential candidate virulence genes has previously been published, which are specific for the human pathogen *F. tularensis*[45]. Surprisingly, analysis of the more complete collection of *Francisella* genomes in the present study reduced this list to only seven *F. tularensis* specific genes. The general lack of specific genes agrees with previous observations that pathogens exhibit specialisation and host-adaption by gene loss rather than gene gain [46]. Thus, a pathogen such as *F. tularensis* should be defined not only by genes that are present but also by those that are lacking.

The seven specific genes in *F. tularensis* were located in three separate gene clusters. The genes in the first cluster (FTT0794, FTT0795, FTT0796) are organised in an operon and were predicted to be involved in exopolysaccharide synthesis. It has been suggested that an exopolysaccharide capsule plays a role in *F. tularensis* virulence [47].

The second gene cluster (FTT1453c, FTT1454c and FTT1458) is present within the *wbt* locus of *F. tularensis*. Genes in this locus encode proteins involved in lipopolysaccharide O-antigen synthesis. The O-antigens of *F. tularensis* contain an insertion element that differentiates it from *F. novicida*[48]. A similar insertion element structure has been reported to be essential for virulence in *Shigella sonnei*[49]. Finally, the last gene cluster contained a single gene, FTT1188, which encodes for a hypothetical membrane protein. This protein does not exhibit significant homology to other proteins in the NCBI non-redundant database and has not yet been characterised in depth. Identification of virulence genes and their role in the virulence of *Francisella* is not straight-forward. Our data suggest that *F. tularensis* virulence cannot be easily explained by a simplistic model which just considers the presence or absence of specific “virulence genes” [36].

*Fa. hongkongensis* and *P. salmonis* both contain many genes that are absent from the *Francisella* genus and are therefore likely to occupy distinctly different ecological niches.

### IS elements

The two principal clades of the *Francisella* genus exhibit several similarities and differences in IS element content and composition. *F. noatunensis* subsp. *noatunensis* contains an expanded set of IS elements similar to those reported for *F. tularensis*. The expanded IS elements in *F. tularensis* are ISFtu1 and ISFtu2, whereas the expanded IS element in *F. noatunensis* is ISFpil. Interestingly, the two subspecies of *F. noatunensis* exhibited contrasting contents of IS elements. While *F. noatunensis* subsp. *noatunensis* contained the highest number of IS elements of all species in the genus, *F. noatunensis* subsp. *orientalis* contains very few IS elements. This may have arisen because of different evolutionary modes in these subspecies.

### Recombination

Recombination is an important process for generating new genetic diversity within bacterial populations and is a known driver of evolution in many bacterial species [50]. The recombination analysis of 45 genomes in the present study confirmed that the level of recombination within the *Francisella* genus is highly variable, ranging from undetectable in *F. tularensis* to moderate in *F. novicida*[36].

We found that the subspecies of *F. noatunensis* exhibited apparently low rates of recombination at levels similar to the human pathogen group of *F. tularensis*. Otherwise, both clade 1 and II displayed moderate recombination rates, largely due to frequent recombination within *F. novicida* (clade 1) and *F. philomiragia* (clade 2). According to our findings, 33% of the core gene set provided evidence of past recombination events, similar in magnitude to estimates for *Campylobacter*[51] and *Neisseria*[52], but higher than previously reported for *Francisella*[36]. It was reported previously that 19.2% of the genes in the core genome of *F. novicida* and *F. philomiragia* have been recombined, but our data suggests that this is could be an underestimate due to a lack of genomic sequences available at that time.

Our findings are similar to reports for other bacterial genera that include highly specialized pathogens. Variable recombination rates have previously been reported for several pathogenic populations, (*Yersina pestis*[53], *Bacillus anthracis*[54] and *Mycobacterium tuberculosis*[55]). It should be noted that if closely related clones of pathogens with low recombination rates are over-represented, this can result in an under-estimation of recombination rates for the entire genus [50,56]. By ensuring proper representation of all subspecies of the *Francisella* genus in the present work, such effects were minimised.

## Conclusions

This work significantly expands the number of sequenced *Francisella* genomes, and therefore provides an important resource for assessing evolutionary and functional relationships within the genus. We identified the core genome in the *Francisella* genus and single nucleotide polymorphisms within these genes were used to infer a robust phylogeny. A high degree of clonality confirmed for *F. tularensis* type A and B, was also identified in *F. noatunensis* subsp. *noatunensis*. Despite infecting different species, it is apparent that these lineages also share certain important evolutionary properties.

Next-generation sequencing enables rapid generation of high quality data for the fast assembly of novel genomes [57]. Growing databases of genome sequences are of significant interest for improving the designs of specific detection assays, conducting epidemiological and ecological studies, microbial forensics [58] and classification of novel bacterial isolates. Comparative genomic approaches together with functional datasets will be critical for the development of assays that specifically target pathogenic *Francisella* strains.

## Methods

### Cultivation and DNA preparation

All strains (Table 1), except FSC845, were cultured on cysteine heart agar plates supplemented with 5–9% chocolatised sheep blood (CHAB) (BD Biosciences, Franklin Lakes, NJ USA), or Modified Thayer-Martin (McLeod) agar plates [59], at 25°C for the eight strains FSC769 to FSC776, and at 37°C for all other strains, in 5% CO_2_ for 5 days (*F. tularensis* strains) or up to ~30 days (non-*F. tularensis* strains).

A loopful of bacteria was suspended in 1 x TRIS-EDTA (TE) buffer to 10^9^ CFU/ml and heat-killed at 95°C for 15 minutes. The bacteria were lysed using Buffer ATL (Qiagen, Hilden, Germany), or similar non-denaturing lysis buffer. DNA was prepared using phenol extraction (PE), or extraction with Biorobot EZ1 (Qiagen, Hilden, Germany) according to the manufacturer’s protocol. Sterility was verified by lack of growth on Modified Thayer-Martin (McLeod) plates [59] after incubation of 10 μl DNA (if PE was used) or lysate (if EZ1 was used) for 10 days.

The strain FSC845 (*Wolbachia persica*) was propagated in cells, and bacteria were collected by differential centrifugation. Bacterial cell pellets were suspended in phosphate-buffered saline (PBS) and DNA was prepared as above. For FSC772 and FDC178, DNA was prepared using the Gentra Puregene Cell kit (Qiagen, Hilden, Germany) and was re-suspended in a hydration solution supplied with the kit. DNA concentrations were measured using Nanodrop (Thermo Scientific, Wilmington, DE, USA).

### Genome sequencing and assembly

DNA for genomic sequencing of 24 isolates from the *Francisella* genus and genetic relatives were prepared as described in Table 1. 19 genomes were sequenced at the National Bioforensic Analysis Center using a 454 FLX machine (454 Life Sequencing Inc., Branford, CT, USA) according to the manufacturer’s instructions. Additional sequencing was performed using the Illumina GS II sequencing 36 bp or 76 bp SE technology. Additional file 4 provides summary data of the sequenced genomes.

Isolates sequenced with both technologies were first assembled using Newbler. Obtained contigs were corrected using the Illumina reads in Nesoni. Isolates sequenced only with the Illumina technology were assembled using Velvet. [60,61].

All 24 isolates included in this study were submitted to IMG/ER [62] for functional annotation. All sequence reads were deposited in Genbank and are freely downloadable from the Genbank project number listed in Table 1.

### 16 S rRNA alignment

The *Francisella* 16 S ribosomal RNA data were obtained using the ribosomal database project [63,64]. The key search words ‘*Francisella*’, ‘*Fangia hongkongensis*’ and ‘*Piscirickettsia salmonis*’ produced 171 hits in the database. After removing duplicates, small sequences and sequences of poor quality, the final dataset consisted of 69 sequences and 1557 bases. To infer the phylogenetic tree of the 16 S data, the software RAxML [65] was used, which is suitable for analysing ribosomal RNA data as covariation patterns are taken into account in the model. The secondary structure was analysed using the substitution model RNA6A by maximising the log-likelihood. The GTR and gamma model was chosen as the standard DNA substitution model. The number of bootstrap replicates was 1000.

### Whole-genome alignment

Multiple genome alignments were computed by employing the progressiveMauve algorithm of the Mauve software [35] using default options. Sequences were extracted and concatenated from each of the 45 genomes to build a core genome of multiple alignments. Core genome SNPs were identified from this alignment as the loci where at least one sequence had a mutated base and it had no missing sequences from any genome included in this study. While it is possible that some of the SNP’s that were specific to a single sequence could have been incorrect due to sequencing errors, this was unlikely in the case of informative sites. Strain-specific SNP’s can have an effect on the estimated value of the mutation rate and the branch length of the phylogenetic tree. However, they do not affect the structure of the tree. The phylogenetic data is deposited in treeBASE [66,67].

### Whole-genome phylogenetic construction

An unrooted neighbour joining tree for the genus *Francisella* with the genetic relatives *Fa. hongkongensis* and *P. salmonis* as an outgroup was constructed using the MEGA5 software [68] based on the numbers of substitutions per nucleotide site in the genome sequences of all strains. The Complete Deletion Method was used for handling alignment gaps.

### Estimation of evolutionary distance

Average nucleotide identity (ANI) was calculated by pairwise genome comparisons with the MUMmer and BLAST algorithms using JSpecies v1.2.1 [69]. A threshold of >95% identity was used to classify genomes as the same species.

### IS element finding

This study focuses on newly sequenced genomes of previously uncharacterized *Francisella* species and subspecies (Additional file 5). Potential IS elements were identified using the BLASTN program (no low-complexity filtering), by querying genome sequences using the ISFinder tool [70] in the IS database and querying flanking regions of the draft genome contigs against the NCBI ‘nt’ database. Altogether, 41 IS elements were found to be present (*e*-value < 10^-2^) in the included genomes. Pairwise BLAST alignments of both left and right flanking regions, and full length IS sequence elements, were constructed for all genomes. The results were evaluated by *e*-value (<10^-3^), identity (90%), bit score (>40) alignment length and proximity to contig ends. Inherently, *de novo* assembly of next-generation sequence data implies problems in resolving repeat structures, resulting in an increased risk of underestimating the IS element copy number.

### Protein orthologs

Forty-five genomes of *Francisella* and two genetic relatives were downloaded from IMG/ER (Table 1 and Additional file 6). Coding sequences were extracted from GenBank files and orthologs were determined using OrthoMCL with a BLAST *e*-value cut-off of 10^-5^ and an inflation parameter of 1.5. We used the OrthoMCL output to construct a table describing genome gene content (Additional file 7) that were used for all pan- and core gene analysis.

Heat maps showing the presence of genes in the *Francisella* genus were generated using custom code in the statistical software R.

### Analysis of recombination

To infer recombination events in the *Francisella* dataset, three recombination detection methods were used: pairwise homoplasy index (Phi) [71], neighbour similarity score (NSS) [72] and maximum chi-square (MaxChi) [73]. These methods are conveniently implemented in the PHI Package [71]. A subset of 742 genes (i.e. the core genome excluding multiple copies of genes) was tested for recombination detection on the entire data as well as on individual lineages in the phylogeny (subspecies populations). It should be mentioned that all recombination methods used here do not correct for multiple testing, which may result in an over-estimation of the presence of recombination, in particular for the full data analysis (comprising 45 isolates in total).

## Abbreviations

ANI: Average nucleotide identity; FLE: *Francisella*-like endosymbionts; MaxChi: Maximum chi-square; NSS: Neighbour similarity score; Phi: Pair wise homoplasy index.

## Competing interests

No competing interests for any of the authors exist.

## Authors’ contributions

AS assembled all genomes and performed analysis of genome and gene content. JA performed the 16 S rRNA alignment, analysis of over-represented COGs and recombination. CO performed the analysis of average nucleotide identity, IS elements and clade specific genes. SD and DJC provided *Francisella* isolates that infect fish and knowledge of fish pathogenic *Francisella*, and contributed to completion of the manuscript. PLA and MF conceived the study. AS, KS, JA, PLI, AJ, PLA, MF drafted the manuscript. All authors read and approved the manuscript.

## Disclaimer

The views and conclusions contained in this document are those of the authors and should not be interpreted as necessarily representing the official policies, either expressed or implied, of the U.S. Department of Homeland Security or the Swedish Civil Contingencies Agency.

## Supplementary Material

Additional file 1 is a table of the average nucleotide identity. Click here for file

Additional file 2 is a figure showing the 16 S rRNA phylogenetic tree. Click here for file

Additional file 3 is a table summarising the recombination results. Click here for file

Additional file 4 is a table of sequencing statistics for included isolates. Click here for file

Additional file 5 is a table of the IS element results. Click here for file

Additional file 6 is a table giving information of public genome. Click here for file

Additional file 7 is the results from orthoMCL. Click here for file
